# Sentinel lymph node mapping with superparamagnetic iron oxide for melanoma: a pilot study in healthy participants to establish an optimal MRI workflow protocol

**DOI:** 10.1186/s12885-022-10146-w

**Published:** 2022-10-14

**Authors:** Loeki Aldenhoven, Caroline Frotscher, Rachelle Körver-Steeman, Milou H. Martens, Damir Kuburic, Alfred Janssen, Geerard L. Beets, James van Bastelaar

**Affiliations:** 1 Department of Surgery, Zuyderland Medical Center, Dr. H. van der Hoffplein 1, 6262 BG Sittard-Geleen, the Netherlands; 2 Present address: Department of Surgery, Zuyderland Medical Center, Postbus 5500 , 6130 MB Sittard, the Netherlands; 3 GROW - School for Oncology and Developmental Biology, Maastricht University, Universiteitssingel 40, 6229 ER Maastricht, the Netherlands; 4 Department of Radiology, Zuyderland Medical Center, Dr. H. van der Hoffplein 1, 6262 BG Sittard-Geleen, the Netherlands; 5 Department of Surgery, Netherlands Cancer Institute - Antoni Van Leeuwenhoek, Plesmanlaan 121, 1066 CX Amsterdam, the Netherlands

**Keywords:** Magnetic iron oxide nanoparticles, Sentinel lymph node biopsy, Melanoma, Surgical oncology

## Abstract

**Background:**

Current pre-operative Sentinel Lymph Node (SLN) mapping using dual tracing is associated with drawbacks (radiation exposure, logistic challenges). Superparamagnetic iron oxide (SPIO) is a non-inferior alternative for SLN mapping in breast cancer patients. Limited research has been performed on SPIO use and pre-operative MRI in melanoma patients to identify SLNs.

**Methods:**

Healthy participants underwent MRI-scanning pre- and post SPIO-injection during 20 min. Workflow protocols varied in dosage, massage duration, route of administration and injection sites. The first lymph node showing a susceptibility artefact caused by SPIO accumulation was considered as SLN.

**Results:**

Artefacts were identified in 5/6 participants. Two participants received a 0.5 ml subcutaneous injection and 30-s massage, of which one showed an artefact after one hour. Four participants received a 1.0 ml intracutaneous injection and two-minute massage, leading to artefacts in all participants. All SLNs were observed within five minutes, except after lower limb injection (30 min).

**Conclusion:**

SPIO and pre-operative MRI-scanning seems to be a promising alternative for SLN visualization in melanoma patients. An intracutaneous injection of 1.0 ml SPIO tracer, followed by a two-minute massage seems to be the most effective technique, simplifying the pre-operative pathway. Result will be used in a larger prospective study with melanoma patients.

**Trial registration:**

ClinicalTrials.gov (NCT05054062) – September 9, 2021.

## Introduction

Sentinel lymph node biopsy (SLNB) is an important prognostic procedure in the staging of malignant melanomas and plays a crucial role in postoperative management [1]. The importance of the SLNB has become even more pronounced since the introduction of adjuvant immunotherapy in stage III melanoma [2]. Previous research showed that SLN locations can be unpredictable and therefore difficult to localize without imaging [3]. The current gold standard for SLN mapping is a dual tracing technique, using a Technetium (^99m^Tc) tracer and Blue Dye [4]. This SLN localization technique, however, is associated with several drawbacks [1]. The major challenge is the short half-life of ^99m^Tc, causing logistic challenges in scheduling SLN mapping (lymphoscintigraphy (LS) and single-photon emission computed tomography (SPECT/CT)) prior to the surgery. Additionally, potential radiation exposure for both patients and health care staff require equipment and protocols for preparation and disposal of radioactive isotopes that are not available in all hospitals [5]. Severe allergic reactions to Blue Dye have been described, in addition to the blue skin discoloration at the injection site of Blue Dye [4, 6, 7].

A novel technique that has been studied in breast cancer patients is superparamagnetic iron oxide (SPIO) tracing, using magnetic nanoparticles [8]. In contrast to isotopes, SPIO nanoparticles have an extended life span [9], providing easier logistics in the preoperative planning. Additionally, SPIO does not carry the risk of radiation exposure to both patients and healthcare staff. Several studies showed that tracing with SPIO is non-inferior to dual tracing with ^99m^Tc and Blue Dye to identify SLNs in breast cancer patients [4, 8, 10–12]. The SPIO tracer Magtrace® (Endomagnetics Ltd, London, UK) is the successor of the Sienna + ® tracer (Endomagnetics Ltd, London, UK) and is a liquid, carboxydextran-coated, non-radioactive lymphatic tracer which can be detected with a handheld localization system (Sentimag®, Endomagnetics Ltd, London, UK) [7]. However, pre-operative scans are still required, as sentinel lymph nodes can be located at several and even unpredictable locations in the human body [3].

SPIO is also expected to be non-inferior to dual tracing in melanoma patients and has previously been analyzed with the use of Sentimag® alone [13, 14]. One small feasibility study published promising preliminary results on pre-operative magnetic resonance imaging (MRI) using SPIO in melanoma patients [15]. Our research team is planning a larger study comparing the diagnostic accuracy of the new technique with SPIO, MRI and the handheld localization system, to the standard dual tracing technique to identify SLNs in melanoma patients. The optimal workflow of the MRI regarding dose of SPIO and route of administration, duration of massage and timing of MRI have however not been defined, with inconclusive results in previous reports [13, 14, 16–18]. Therefore, the aim of this study was to develop an optimal SPIO-MRI workflow protocol in healthy participants to localize SLNs.

## Materials and methods

### Participants

In August 2021, healthy participants were enrolled in a small pilot study, performed at Zuyderland Medical Center, the Netherlands. Since this is a pilot study, no sample size calculation was performed. Participants were enrolled until a feasible MRI workflow protocol was defined. The study adopted a step-by-step approach in which the information on the results of the pre-operative MRI obtained in the first participants leads to an optimalization of the workflow protocol of the following participants. Exclusion criteria were known intolerance/hypersensitivity to iron or dextran compounds and the standard MRI exclusion criteria [19]. Participants provided written consent upon participation. This pilot study was approved by the institutional ethics committee (METC Zuyd, Z2021082) and registered at ClinicalTrials.gov (NCT05054062).

### SPIO and MRI

MRI was performed with a Magnetom Avanto fit 1.5 T system from Siemens and an 18-channel body coil. Magtrace® (Endomagnetics Ltd, London, UK) was used as SPIO tracer and is a black-brownish sterile aqueous suspension consisting of carboxydextran-coated particles with a particle size of 60 nm. Magtrace® contains circa 28 mg iron per ml and is intended for subcutaneous use [16, 20, 21].

### MRI protocol

Participants were placed in the scanner in supine position with arms parallel to the body. Pre- and post-SPIO images were obtained using a T_1_-weighted volumetric interpolated breath-hold examination Dixon sequence (*fl3d2, repetition time / echo time 6.64/4.77 ms; field of view 46.2 cm; Bandwidth 580 Hz; slice thickness 2 mm; imaging time 30 s.). Dependent on participants’ length, injection site and potential SLN regions [3], body parts were separately scanned pre-SPIO. Subsequently, SPIO was injected sub- or intracutaneously followed by a massage, while the participant was still positioned in the scanner. Post-SPIO images were obtained and merged to whole-body scan images. Images were analyzed by two radiologists using a Picture Archiving and Communication System workstation (Sectra). SPIO uptake from the injection sites and the migration through lymphatic vessels to SLNs was assessed. The first appearing lymph node (LN), showing susceptibility artefacts of SPIO accumulation, was considered to be the SLN. The LN regions were identified.

### Step-by-step approach

**Fig. 1:**
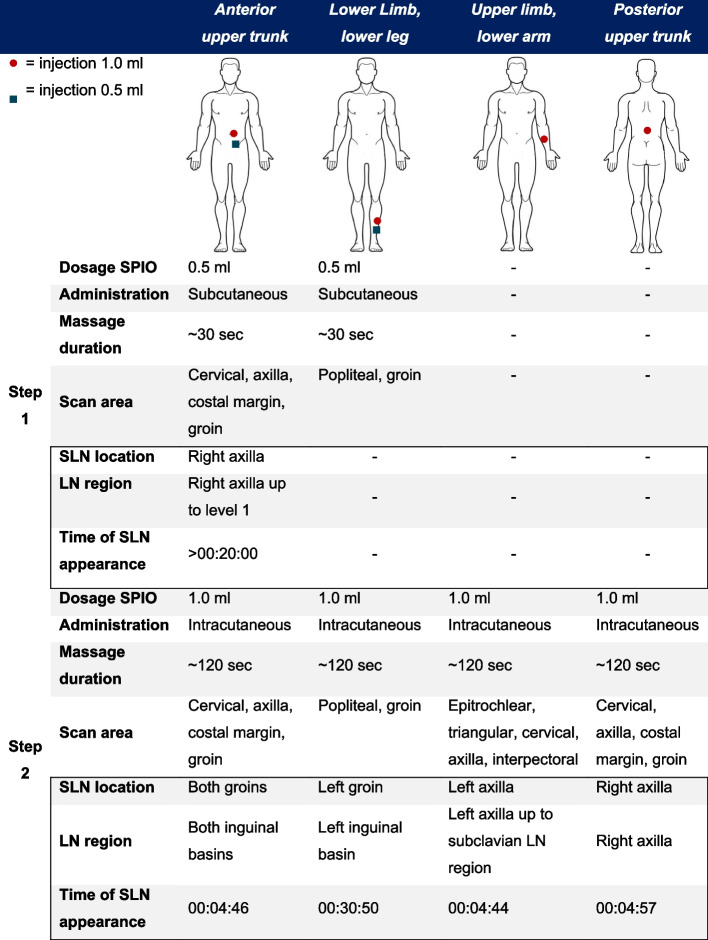
Detailed information about SPIO injections and time of appearance of SLNs

In order to find the optimal workflow protocol, the injection sites, dosages of Magtrace®, massage duration and route of administration varied among protocols. The first two participants, anterior upper trunk and lower limb, received a subcutaneous injection with 0.5 ml SPIO suspension, followed by a 30 s vigorous massage. First post-injection imaging was performed approximately two minutes after injection. Every other minute, images were produced and repeated until 10 min, then, every two minutes until 20 min. When after 20 min no susceptibility artefacts in LN regions were noted on MRI, scanning was repeated one hour post-contrast. Based on the findings of the first two participants, the protocol for the next four participants was changed with higher dose of 1.0 ml SPIO suspension, with an intracutaneous injection rather than subcutaneous (anterior and posterior trunk, lower arm and lower leg), and with a longer two-minute vigorous massage. Post-injection MR imaging was performed at the same time intervals as in the first two participants. Eighteen days post-injection, images of SPIO distribution to other LNs/regions was evaluated. Detailed information about SPIO location, dosage, massage duration, route of administration and scan area per injection site can be found in Fig. 1 in the results section.

## Results

### Participants and artefacts

All six participants were healthy and five out of six were male. The mean age of the participants was 37.5 years (range 27—61 years). An artefact at the injection site could be identified on T_1_-weighted post-contrast images in all participants. An artefact in SLNs was identified in five of six participants. In four participants (1.0 ml SPIO), the lymphatic tract could be identified from injection site up to the LN. Participant characteristics can be found in Table 1.

**Table 1:** Participant characteristics

	**Participant**					
Age (years)	42	61	29	34	27	32
Sex	M	M	F	M	M	M
Height (cm)	184	172	161	185	172	180
Weight (kg)	91	71	44	83	80	71
BMI (kg/m^2^)	26.9	24.0	17.0	24.3	27.0	21.9
Injection site	Ant. upper trunk	Lower limb, lower leg	Upper limb, lower arm	Ant. upper trunk	Lower limb, lower leg	Post. upper trunk

*M* Male, *F* Female, *BMI* Body Mass Index, *Ant* Anterior, *Post* Posterior

### Time of appearance of LNs

A summary of the time of lymphatic uptake and affected LN regions is shown in Fig. 1. After subcutaneous injection of 0.5 ml SPIO (anterior upper trunk and lower leg), no artefacts were identified in LN regions in the first 20 min. At one hour post-injection, SPIO injected in the upper trunk in participant one drained to the right axillary region. In participant two no artefacts were identified after 0.5 ml SPIO injection in the lower leg. A 1.0 ml SPIO injection administered in the lower arm in participant three drained into the left axillary region. The anterior and posterior trunk of participants four and six drained into both groins and right axillary region, respectively. All artefacts were observable on the MRI within five minutes. At the late scan at 18 days there were other LNs in the same region with susceptibility artefacts, without artefacts in other LN regions. Regarding the lower leg location, no artefacts in LNs were seen with a 0.5 ml SPIO subcutaneous injection in participant two, whereas with the 1.0 ml dose intracutaneously in participant five SPIO uptake was observed after 30 min in the left groin.

### Images

**Fig. 2:**
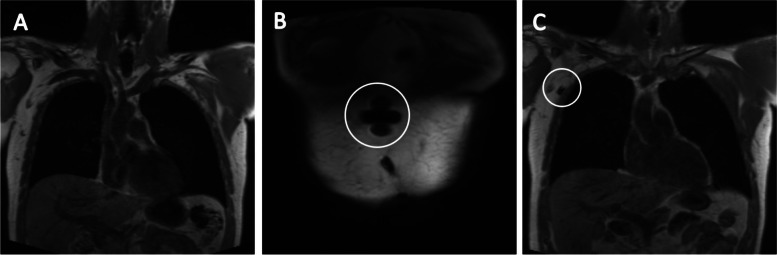
Pre- and post-injection (0.5 ml) T_1_-weighted coronal images of the anterior upper trunk in participant one. **A** Image before tracer injection. **B** Image of injection site showing a large decrease in signal. **C** Image of lymphatic tracer uptake in right axillary LN, one hour post-injection. The first LN showing a large decreased signal and therefore considered to be the SLN, indicated with the white circle

**Fig. 3:**
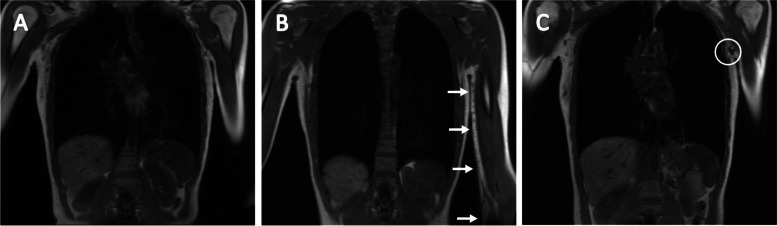
Pre- and post-injection (1.0 ml) T_1_-weighted coronal images of the upper limb, lower arm. **A** Image before tracer injection. **B** Image of injection site showing a large decrease in signal in LN tract, indicated with the white arrows. **C** Image of lymphatic tracer uptake in right axillary LN, five minutes post-injection. The first LN showing a large decreased signal and therefore considered to be the SLN, indicated with the white circle

**Fig. 4:**
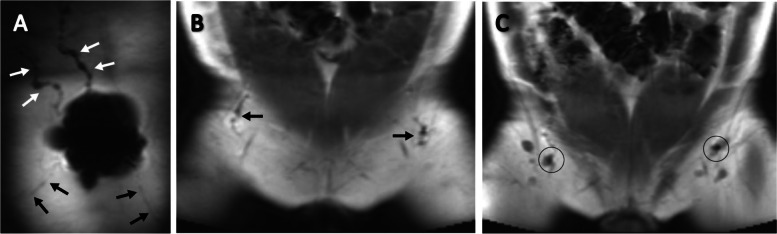
Post-injection (1.0 ml) T_1_-weighted coronal images of the anterior upper trunk. **A** Image after tracer injection. Lymphatic uptake towards upper body is indicated with the white arrows and lymphatic uptake towards groins is indicated with the black arrows. **B** Image showing a large decrease in signal in groin lymph vessels. **C** Image of lymphatic tracer uptake in both groin LNs, five minutes post-injection. The first LN showing a large decreased signal and therefore considered to be the SLN, indicated with the black circle

Images showing a susceptibility artefact due to SPIO uptake in lymphatic vessels and LNs are shown in Figs. 2, 3 and 4. Observable artefacts per injection site are indicated with arrows and circles.

### Side effects

**Fig. 5:**
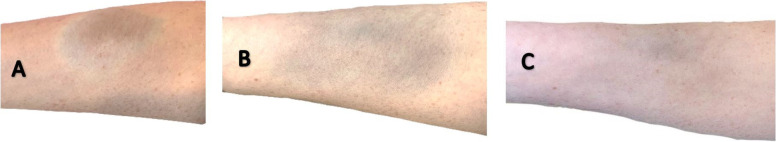
Skin discoloration of the lower arm after 1.0 ml intracutaneous SPIO injection. **A** Two hours post-injection. **B** Five weeks post-injection. **C** Five months post-injection

Skin discoloration at the injection site was noted in the four participants who received the 1.0 ml intracutaneous injection (Fig. 5). Susceptibility artefacts on MRI persists for a longer period of time.

## Discussion

This pilot study developed a feasible and safe pre-operative SPIO-MRI workflow protocol in healthy subjects. A dose of 1.0 ml SPIO is injected intracutaneously while the patient is positioned in the scanner, followed by a two-minute vigorous massage. MRI scanning commences within five minutes of intracutaneous injection. This SPIO-MRI workflow protocol can be used in subsequent studies comparing this new SLN mapping technique with the current gold standard of ^99m^Tc and Blue Dye in melanoma patients.

Previous studies focused on the “comparison” between SLN identification with SPIO (whether or not in combination with MRI) and dual tracing with isotopes and LS + SPEC/CT. However, workflow protocols varied among studies. Dosage of SPIO varied between 0.5 ml and 2 ml, whether or not diluted with saline up to 5.0 ml, route of administration was either described as subcutaneous or intracutaneous and massage duration ranged between no massage and 20 min. Moreover, MRI timing between pre- and post-injection imaging varied between protocols [12–14, 16–18].

In a previous study, the dosage did not affect the identification of SLNs, nor the time to first appearance of SLNs [18]. The present study showed that the dosage of SPIO (0.5 ml vs. 1.0 ml) affected the SLN identification. A 0.5 ml subcutaneous injection with SPIO did not lead to SLN identification in one of two participants. However, in the present study a lengthened time of SPIO migration to SLNs in the lower dosage was observed. In the second participant, an artefact was only observed after one hour. In contrast with the latter, 1.0 ml intracutaneous injection showed immediate uptake and SPIO distributed to the SLN within 5 min. Similar to SPIO volume, distance from injection site to SLNs tends to affect the time of first appearance of SLNs. Time of lymphatic uptake of SPIO injected in the participants’ trunk or upper limb differed from time of lymphatic uptake of SPIO injected in the lower limb. This was 5 min versus 30 min, respectively. Migration time of SPIO may therefore differ, depending on the location of the injection site. Findings of time to lymphatic SPIO uptake are consistent with clinical experience in breast cancer patients (uptake within 5 min). Lymphatic drainage in.

the lower limb might be improved by active movement of the leg. This will be investigated further in a planned larger prospective study with melanoma patients.

Previous studies have shown that SPIO is still detectable 30 days post injection. Consistent with our findings, SPIO artefacts were still clearly observable on MRI 18 days post injection [18, 22, 23]. However, no distinction could be made between the SLNs and other LNs on MRI 18 days after SPIO injection. Detection with the handheld magnetometer was not performed after 18 days, as this was not part of the research objective. Based on previous research and current findings, extending the timeframe to seven days (from SPIO injection to SLN harvesting) as described by the Magtrace® information documents [20, 21], seems feasible and beneficial for pre-operative planning and eliminating logistic challenges.

Skin discoloration might be considered a drawback, however, discoloration tends to vanish over time and the injection site is mainly excised as part of WLE in patients with melanoma.

As this is a small feasibility study, the limited number of included participants was insufficient to perform statistical analyses.

## Conclusion

This pilot study established a pre-operative SPIO-MRI workflow protocol in healthy volunteers, a protocol that can be used to identify SLNs in patients with melanoma. An intracutaneous injection of 1.0 ml SPIO tracer, followed by a two-minute massage and MRI scanning after 5 min is an effective technique. The value of this technique to identify SLNs in patients with melanoma should be studied first in a prospective feasibility study in patients. This seems to be a promising alternative for SLN visualization in patients with melanoma. The next step is to start a feasibility study in patients with melanoma using this MRI workflow protocol.

## Data Availability

All data generated or analyzed during this study is included in this published article.
